# Imipenem/cilastatin/relebactam dosing regimen justification using exposure–efficacy analyses in participants with hospital-acquired bacterial pneumonia or ventilator-associated bacterial pneumonia in the RESTORE-IMI 2 phase 3 study

**DOI:** 10.1128/aac.01313-25

**Published:** 2026-01-26

**Authors:** Munjal Patel, Garrett Nieddu, C. Andrew DeRyke, Katherine Young, Luke Francis Chen, Amanda Paschke, Matthew L. Rizk, Ferdous Gheyas

**Affiliations:** 1 Merck & Co., Inc. 2793 , Rahway, New Jersey, USA; Providence Portland Medical Center, Portland, Oregon, USA

**Keywords:** exposure–efficacy, imipenem, nosocomial pneumonia, relebactam, pharmacokinetics

## Abstract

An exposure–efficacy analysis of the phase 3 RESTORE-IMI 2 study (NCT02493764) evaluated the relationship between plasma exposure of imipenem and relebactam and key efficacy endpoints (primary: 28-day all-cause mortality [ACM]; secondary: clinical response at early follow-up [EFU; 7–14 days after the end of treatment]) in adult participants with hospital-acquired bacterial pneumonia (HABP) or ventilator-associated bacterial pneumonia (VABP) who received imipenem/cilastatin/relebactam 500/500/250 mg (IMI/REL). Participants from the IMI/REL treatment group in the microbiological-modified intent-to-treat population who had pharmacokinetic (PK) data and relevant baseline pathogen susceptibility data available were included (*N* = 211). Previously developed population PK models were used to derive steady-state imipenem and relebactam exposures. Exposures were analyzed against the primary and secondary endpoints overall and by the most common key pathogens identified during RESTORE-IMI 2 (*Pseudomonas aeruginosa*, *Klebsiella pneumoniae*, *Acinetobacter calcoaceticus–baumannii* complex, and *Escherichia coli*). Most participants (82.0%; *n* = 168/205) achieved the imipenem exposure target, and 83.9% (*n* = 177/211) achieved the relebactam exposure target. ACM was 16.1% overall; 11% among participants who met the imipenem exposure target, and 18% among those who met the relebactam exposure target. At EFU, 62.1% of participants experienced a favorable clinical response. Efficacy was similar across the overall exposure range and by key pathogens. There were no apparent trends in ACM rates by imipenem or relebactam exposure distributions overall or by individual key pathogens, suggesting an exposure–efficacy plateau. These results further support the recommended and currently approved IMI/REL 500/500/250 mg dosing regimen for patients with HABP/VABP.

## INTRODUCTION

Hospital-acquired bacterial pneumonia (HABP) and ventilator-associated bacterial pneumonia (VABP) are common nosocomial infections, with mortality rates of 20–50% (1). The prevalence of multidrug-resistant (MDR) *Pseudomonas aeruginosa* isolates and extended-spectrum β-lactamase (ESBL)-producing *Enterobacterales* isolates in patients with HABP or VABP can exceed 30% depending on the patient population and region (2–4). The risk of antibacterial treatment failure is increased in the presence of MDR pathogens, exacerbating the already high morbidity and mortality rates in patients with HABP and VABP (5). To improve patient survival, appropriately timed and effective antibacterial agents that combat MDR pathogens are required (6). The combination of the β-lactamase inhibitor relebactam (REL) plus the carbapenem imipenem demonstrates broad *in vitro* activity against MDR gram-negative pathogens (7–9). REL influences the antibacterial activity of imipenem by inhibiting class A and C β-lactamases expressed in the pathogen, thereby protecting imipenem from degradation (10). The novel β-lactam–β-lactamase inhibitor combination of imipenem/cilastatin 500/500 mg (IMI) and REL 250 mg (IMI/REL) has shown a favorable safety profile in patients (11–14), as well as efficacy in treating serious infections caused by *Klebsiella pneumoniae* carbapenemase- or ESBL-producing *Enterobacterales*, and carbapenem-resistant/AmpC β-lactamase-producing *P. aeruginosa* (13, 14). In the phase 3 RESTORE-IMI 1 study, IMI/REL was found to be efficacious and well tolerated in hospitalized patients with HABP/VABP, complicated intra-abdominal infection (cIAI), or complicated urinary tract infection (cUTI) caused by imipenem-nonsusceptible (but colistin- and imipenem/REL-susceptible) pathogens (14). The phase 3 RESTORE-IMI 2 study explored the clinical efficacy of IMI/REL further in adults with HABP/VABP caused by gram-negative pathogens and compared IMI/REL with piperacillin/tazobactam (PIP/TAZ); 28-day all-cause mortality (ACM) was 15.9% with IMI/REL and 21.3% with PIP/TAZ (difference: −5.3%; 95% CI: −11.9 to 1.2) in the modified intent-to-treat (MITT) population (11). Clinical response rates at early follow-up (EFU) were 61.0 and 55.8% for IMI/REL and PIP/TAZ, respectively (difference: 5.0%; 95% CI: −3.2 to 13.2) (11). Based in part on the results of these clinical trials, the fixed-dose combination of 500/500/250 mg IMI/REL, with dose adjustments for renal impairment, was approved for the treatment of patients with cUTI, cIAI, and HABP/VABP (12, 13).

The clinical benefit of effective antibacterial agents is dependent on both the timing of treatment and the appropriate dosage. Critically ill patients receiving antibacterial treatments may experience altered and variable pharmacokinetics (PK) of the respective drug. Therefore, a comprehensive understanding of how various factors affect PK is of scientific interest in the field of antibacterial clinical pharmacology (15–19). Appropriate dosage of a drug depends, in part, on PK and the pharmacodynamics (PD) of a drug; knowledge of these factors can provide insight into optimal dosing regimens across diverse patient populations, including special populations, such as critically ill patients with HABP or VABP (20). The objective of the analysis reported here was to assess the relationship between exposure and efficacy in patients with HABP/VABP based on data from participants in the RESTORE-IMI 2 study.

## MATERIALS AND METHODS

### RESTORE-IMI 2 study design

RESTORE-IMI 2 (ClinicalTrials.gov identifier: NCT02493764; protocol MK-7655A-014) was a phase 3, randomized, controlled, double-blind, non-inferiority study that compared the efficacy and safety of IMI/REL with PIP/TAZ in adults with HABP/VABP (11). The study design and primary findings have been published elsewhere (11). The study was conducted in accordance with the principles of Good Clinical Practice and approved by the appropriate institutional review boards and regulatory agencies.

The full inclusion and exclusion criteria have been previously reported (11). In brief, participants were ≥18 years of age, required intravenous antibacterial therapy for non-ventilated HABP, ventilated HABP, or VABP, and provided a lower respiratory tract specimen within 48 h of screening. Participants included in this analysis received a dose of 500/500/250 mg IMI/REL administered intravenously every 6 h for 7–14 days (the currently approved regimen) (12, 13). Dosing regimens were adjusted in participants with renal impairment (12, 13). Sparse blood samples for PK assessments were obtained at screening and approximately 0.5 and 4 h post-dose on Days 1, 3, and 6 (over the 7–14-day dosing period) (11). Samples collected at a later infusion within 24 h of Day 3 or Day 6 were acceptable if it was not feasible to collect blood samples post-dose on those days. Due to operational reasons, some participants may not have had samples taken at some time points. The actual time of sampling was used in the population PK (popPK) analysis (21).

The primary endpoint of RESTORE-IMI 2 was 28-day ACM in the MITT population, defined as all randomized participants who received ≥1 dose of study drug and whose baseline Gram stain did not show only gram-positive cocci (11). The key secondary endpoint was clinical response at EFU, defined as 7–14 days after the end of treatment in the MITT population. The microbiological MITT (mMITT) population comprised participants from the MITT population with ≥1 baseline lower respiratory tract pathogen species against which imipenem/REL is known to have antibacterial activity (11). Favorable clinical response was considered a cure or sustained cure. Cure was defined as the resolution of all pre-therapy signs and symptoms of the index infection (or return to ‘pre-infection status’) and no need for additional antibacterial therapy for the index infection. Sustained cure was defined as cure at prior visit, with no evidence of resurgence at the EFU or Day 28 post-randomization visit. Previously developed popPK models were used to estimate steady-state exposures for imipenem and REL (21).

### Exposure–efficacy analysis population

The exposure–efficacy analysis population comprised participants from the mMITT population who had ≥1 evaluable PK measurement (11).

### Exposure–efficacy analysis methods

The relationship between exposure and efficacy was assessed overall and by the presence of one of four key pathogens (*P. aeruginosa*, *K. pneumoniae*, *Acinetobacter calcoaceticus–baumannii* complex, and *E. coli*). The PK/PD target for REL was a ratio of at least eight for free plasma drug area under the concentration–time curve from 0 to 24 h (*f*AUC_0–24_) over the minimum inhibitory concentration (MIC; *f*AUC_0–24_/MIC) (10, 21). For the analysis of imipenem, the PK/PD target was at least 40% of the dosing interval with a free plasma drug concentration that exceeded the MIC (%*f*T > MIC) (21). For each participant, exposure endpoints were derived using individual empirical Bayesian estimates of PK parameters (e.g., clearance, volume of distribution in the central compartment, and volume of distribution in the peripheral compartment): *f*AUC_0–24_/MIC was calculated for REL using the unbound fraction of 0.78 and the pathogen-specific MIC at baseline, whereas %*f*T > MIC was calculated for imipenem using the unbound fraction of 0.80 and the pathogen-specific MIC at baseline. The MIC used in the analysis was the imipenem MIC measured in the presence of 4 μg/mL of REL since REL itself does not possess intrinsic antibacterial activity (10). The REL concentration of 4 µg/mL was selected based on the average unbound human plasma concentration after multiple REL doses (22). In cases of polymicrobial infection, with multiple MIC values available, the highest MIC value was used.

An exploratory analysis was conducted to assess the relationship between the above-described drug exposures and efficacy endpoints plotted by quartiles for individual REL (*f*AUC_0–24_/MIC) and imipenem (%*f*T > MIC) exposure measurements.

All analyses were conducted in R software (version 4.3.2) using descriptive statistics.

## RESULTS

### Participants

**Fig 1:**
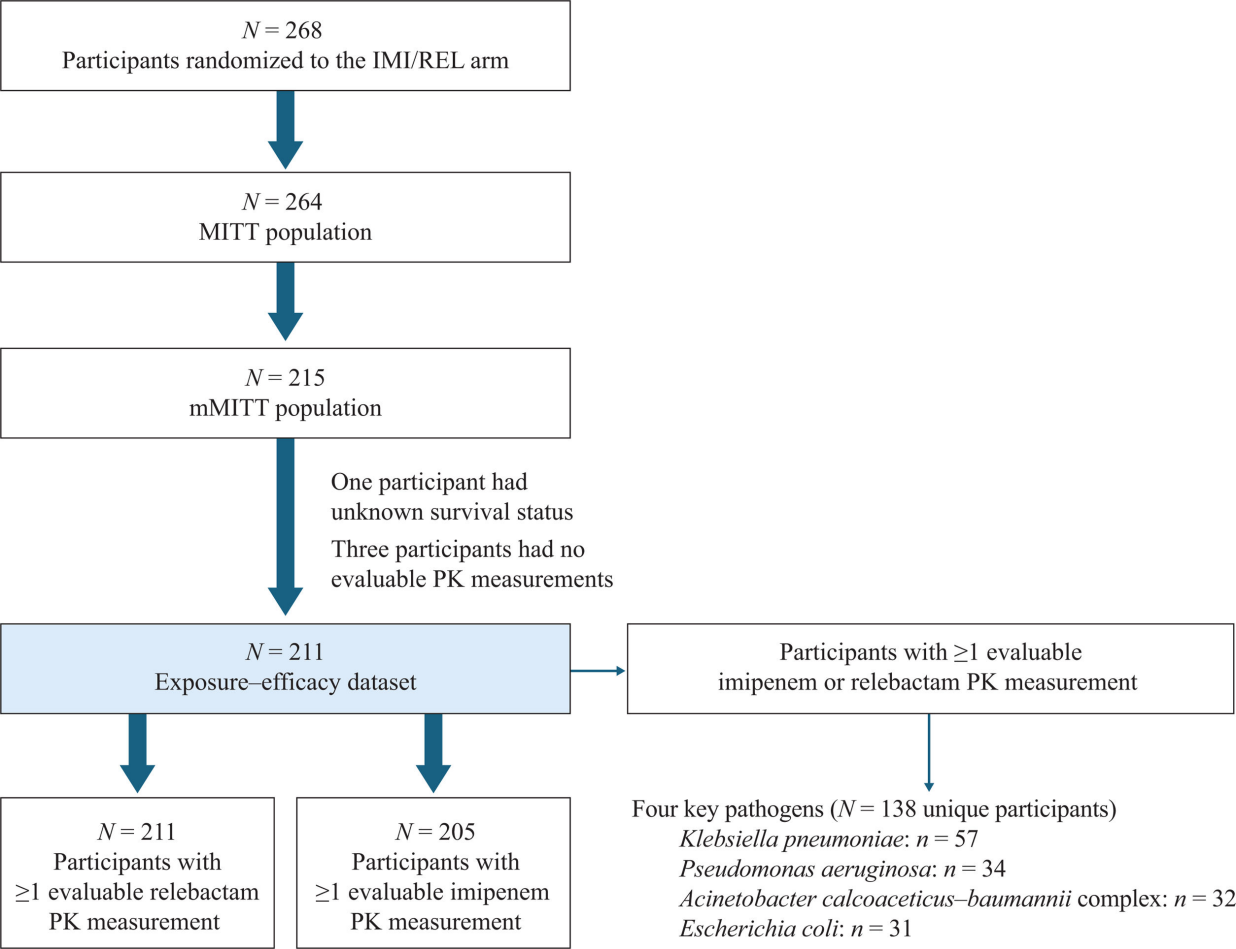
Participant population for the exposure–efficacy analysis. MITT, modified intent-to-treat; mMITT, microbiological modified intent-to-treat; PK, pharmacokinetic. Note that some participants had polymicrobial infections, and, thus, the number of participants with any key pathogen infection is less than if summing the number with each individual pathogen.

The source data set for the exposure–efficacy analyses consisted of the 268 participants randomized to receive IMI/REL in RESTORE-IMI 2, of whom 264 participants were included in the MITT population and 215 in the mMITT population (Fig. 1) (11). The exposure–efficacy analysis population used for the current analysis (*n* = 211) excluded four participants in the mMITT population who lacked data for survival status (*n* = 1) or an evaluable PK measurement for imipenem and REL (*n* = 3). REL exposure–efficacy analyses were performed on 211 participants. A further six participants who were included in the overall exposure–efficacy analysis population lacked an evaluable imipenem PK measurement; therefore, imipenem exposure–efficacy analyses were performed in a population of 205 participants.

The key pathogens included in the current analysis were *K. pneumoniae*, *P. aeruginosa*, *A. calcoaceticus–baumannii* complex, and *E. coli*, which were the most common pathogens identified in the primary analysis (11) and among those species included in the IMI/REL label whose isolates have been shown to be susceptible to IMI/REL both *in vitro* and in clinical infections (13). Among the exposure–efficacy analysis population, approximately two-thirds of participants had at least one key pathogen present (total of 138 participants), with *K. pneumoniae* isolated from 57 participants, *P. aeruginosa* from 34 participants, *A. calcoaceticus–baumannii* complex from 32 participants, and *E. coli* from 31 participants (Fig. 1).

Most participants in the exposure–efficacy analysis population were White (79.6%) and male (71.6%; Table 1). The mean age was 59.5 years; 41.7% were ≥65 years of age, and 21.3% were ≥75 years of age.

**TABLE 1:** Baseline characteristics of the exposure–efficacy analysis population

Characteristic	Participants (*N* = 211)
Age	
Mean age, years (SD^*a*^)	59.5 (17.8)
Median age, years (range)	62.0 (18.0–96.0)
Age group, *n* (%)	
<65	123 (58.3)
≥65	88 (41.7)
≥75	45 (21.3)
Male sex, *n* (%)	151 (71.6)
Body weight	
Mean, kg (SD^*a*^)	77.0 (19.6)
Median, kg (range)	76.2 (26.8–151)
Race, *n* (%)	
White	168 (79.6)
Asian	31 (14.7)
Other	9 (4.3)
Black	3 (1.4)

^*a*^ SD, standard deviation.

### Overall efficacy

In the exposure–efficacy analysis population, the overall rate of 28-day ACM was 16.1% (*n* = 34/211). For clinical response at the EFU endpoint (mMITT population), a total of 211 participants were available for the analysis. A favorable EFU clinical response was seen in 62.1% of participants (*n* = 131/211), a response rate similar to that observed in the primary analysis of the MITT population (11). When considering individual key pathogens, favorable EFU clinical response ranged from 36.3% for *P. aeruginosa* to 61.3% for *E. coli*. Furthermore, rates of 28-day ACM and clinical cure rates at EFU stratified by baseline imipenem/REL MIC are presented in Table S1.

### Exposure–efficacy results

#### Relebactam

In the exposure–efficacy analysis population, the overall rate of 28-day ACM with REL was 16.1% (*n* = 34/211). For clinical response at the EFU endpoint, a total of 211 participants were available for the analysis (mMITT population), of whom 131 (62.1%) had a favorable EFU clinical response (Table 2).

**TABLE 2:** Clinical response by achievement of the relebactam exposure target (*f*AUC_0–24_/MIC ≥ 8) and/or imipenem exposure target (%*f*T > MIC for at least 40% of the dosing interval)^*a*^^*,^b^*^

	Participants with clinical response at early follow-up (7–14 days after the end of treatment)					
	Relebactam exposure (*f*AUC_0–24_/MIC)			Imipenem exposure (%*f*T > MIC)		
	<8	≥8	Overall	<40%	≥40%	Overall
Overall, *n* (%)	21/34 (61.8)	110/177 (62.1)	131/211 (62.1)	24/37 (64.9)	104/168 (61.9)	128/205 (62.4)
By pathogen, *n* (%)						
*Pseudomonas aeruginosa*	2/2 (100)	12/32 (37.5)	14/34 (41.1)	0/3 (0)	11/30 (36.6)	11/33 (36.3)
*Klebsiella pneumoniae*	NA	34/57 (59.6)	34/57 (59.6)	NA	34/56 (60.7)	34/56 (60.7)
*Acinetobacter calcoaceticus–baumannii* complex	14/24 (58.3)	5/8 (62.5)	19/32 (59.4)	14/24 (58.3)	3/4 (75)	17/28 (60.7)
*Escherichia coli*	NA	19/31 (61.3)	19/31 (61.3)	NA	18/30 (60.0)	18/30 (60.0)

^*a*^ The data are by per pathogen per participant for the pathogen summaries. When participants had multiple pathogens, the highest MIC was used for the overall summary.

^*b*^ *%f*T > MIC, percentage of time free drug concentration exceeded the minimum inhibitory concentration; *f*AUC_0–24_/MIC, free area under the concentration-time curve from 0 to 24 h normalized to MIC; MIC, minimum inhibitory concentration; NA, not available.

**Fig 2:**
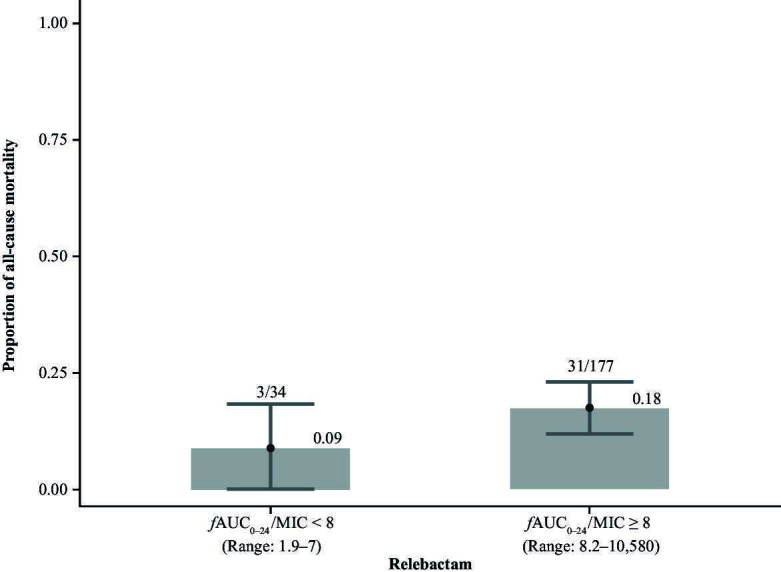
All-cause mortality by achievement of the relebactam exposure target (*f*AUC_0–24_/MIC ≥ 8) in the exposure–efficacy analysis population (*n* = 211). AUC, area under the concentration-time curve; *f*AUC_0–24_/MIC, free area under the concentration-time curve from 0 to 24 h normalized to MIC; MIC, minimum inhibitory concentration.

**Fig 3:**
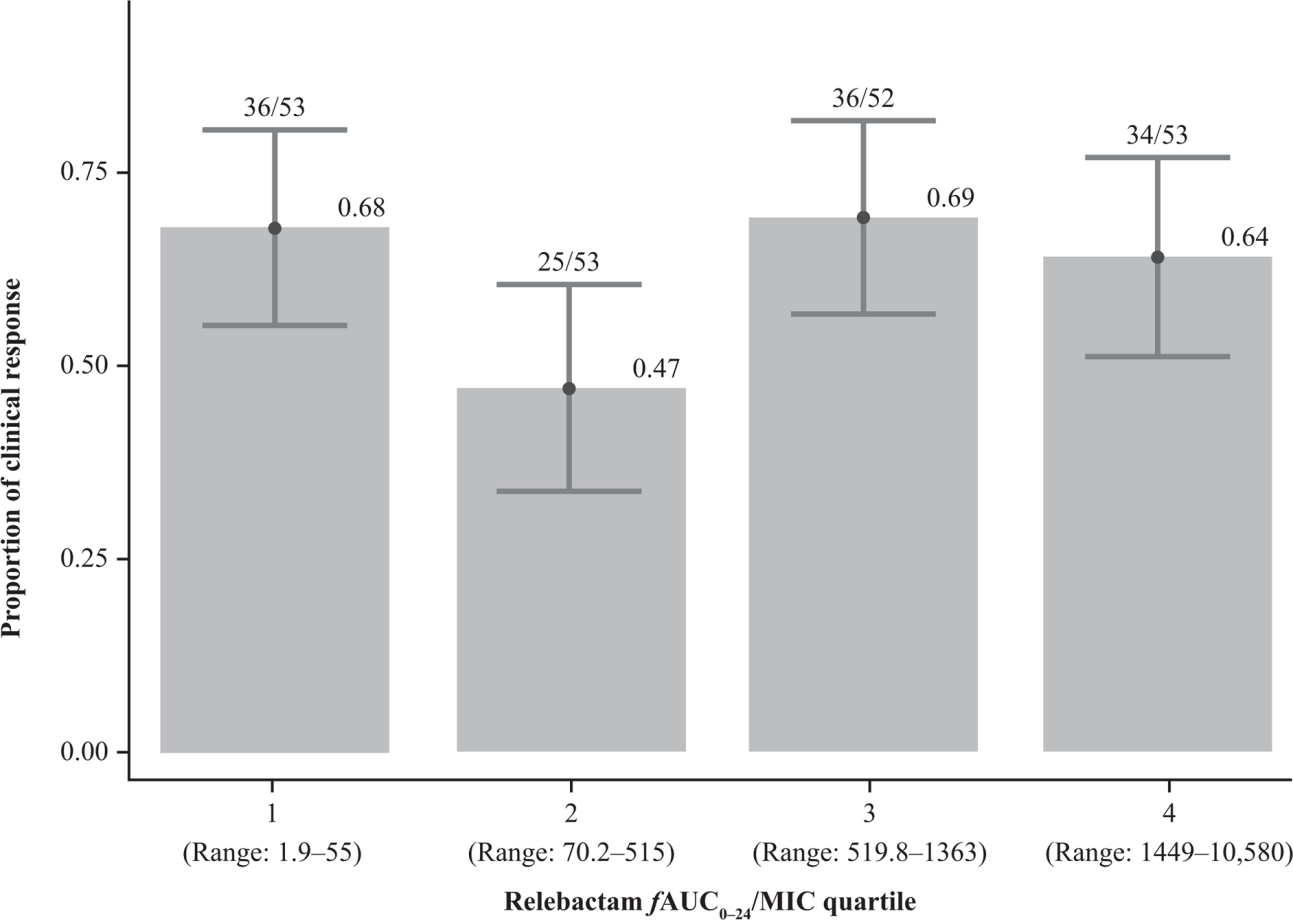
Clinical response by relebactam exposure. AUC, area under the concentration-time curve; *f*AUC_0–24_/MIC, free area under the concentration-time curve from 0 to 24 h normalized to MIC; MIC, minimum inhibitory concentration.

Most participants (83.9% [*n* = 177/211]) achieved the REL exposure target ratio of ƒAUC_0–24_/MIC ≥ 8. The median ƒAUC_0–24_/MIC for REL was 697.7 for the overall population, ranging from 4.4 for *A. calcoaceticus–baumannii* complex to 1540.6 for *E. coli* (Table 3). The 28-day ACM rate was 18% (31/177) among participants who achieved the target and 9% for those who did not (3/34). There was no apparent relationship between 28-day ACM and REL ƒAUC_0–24_/MIC ratio when considering all participants (Fig. 2) or considering by pathogen (Fig. S1). There was also no apparent trend between clinical efficacy at EFU and REL *f*AUC_0–24_/MIC ratio among all participants; 62.1% (110/177) who met the exposure target achieved clinical efficacy at EFU versus 61.8% (21/34) of those who did not (Table 2). No trend in clinical efficacy at EFU was apparent when REL exposure was assessed by quartiles (Fig. 3); thus, a formal modeling analysis was not conducted.

**TABLE 3:** Summary statistics of PK/PD exposures stratified by the key baseline pathogen type^*a*^^,^^*b*^

Variable	Statistic	*Pseudomonas aeruginosa* (*n* = 34)	*Klebsiella pneumoniae* (*n* = 57)	*Acinetobacter calcoaceticus–baumannii* complex (*n* = 32)	*Escherichia coli* (*n* = 31)	Overall (*N* = 138)
*f*AUC_0–24_/MIC relebactam	Median (range)	385.9 (1.9–8,715.4)	1,047.0 (55.0–4,378.8)	4.4 (1.9–788.5)	1,540.6 (656.5–5,231.6)	697.7 (1.9–8,715.4)
	IQRs	(1.9–171.2)	(55.0–452.1)	(1.9–2.7)	(656.5–1,105.2)	(102.3–1,499.4)
*%fT* > MIC imipenem	Median (range)	99.7 (0–99.7)	99.7 (0–99.7)	0 (0–99.7)	99.7 (0–99.7)	99.7 (0–99.7)
	IQRs	(0–85.2)	(0–99.7)	(0–0)	(0–99.7)	(66.5–99.7)

^*a*^ The analysis set presented in this table is a subset of the exposure–efficacy analysis data set.

^*b*^ *%f*T > MIC, percentage of time free drug concentration exceeded the minimum inhibitory concentration. IQR, interquartile range; *f*AUC_0–24_/MIC, free area under the concentration-time curve from 0 to 24 h normalized to MIC; MIC, minimum inhibitory concentration.

#### Imipenem

In the exposure–efficacy analysis population, the overall rate of 28-day ACM with imipenem was 15.6% (*n* = 32/205). For clinical response at the EFU endpoint, a total of 205 participants were available for the analysis (mMITT population), of whom 128 (62.4%) had a favorable EFU clinical response (Table 2).

**Fig 4:**
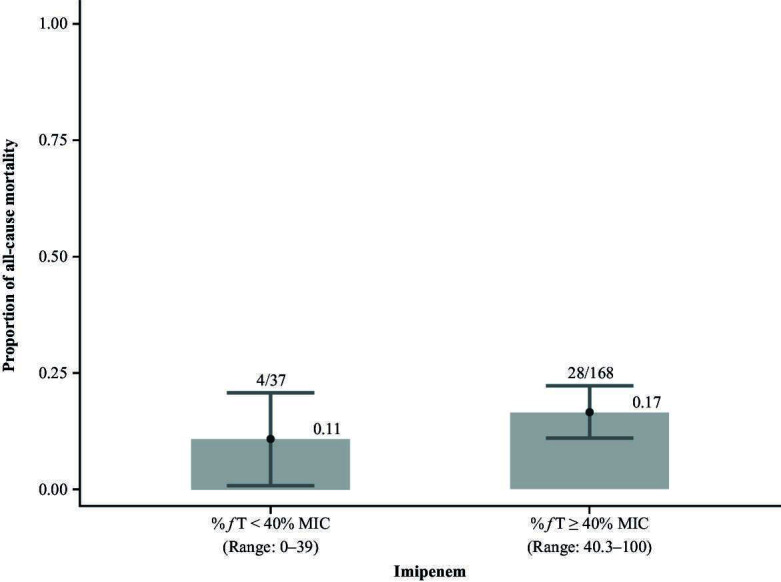
All-cause mortality by achievement of target imipenem exposure (*f*T > MIC for at least 40% of the dosing interval) in the exposure–efficacy analysis population (*n* = 205). %*f*T > MIC, percentage of time free drug concentration exceeded the minimum inhibitory concentration.

Most participants (82.0% [*n* = 168/205]) achieved the imipenem PK exposure target (*ƒ*T exceeded the MIC for at least 40% of the dosing interval). The median %*ƒ*T > MIC of dosing interval time was 99.7% for the overall population, ranging from 0% for *A. calcoaceticus–baumannii* complex to 99.7% for all three of the other pathogens (Table 3). There were no apparent trends in 28-day ACM rates when considering participant groups in which imipenem exposure did versus did not meet the exposure target (Fig. 4), including when stratified by key pathogens (Fig. S2). No trends in 28-day ACM rates were apparent when imipenem exposure was assessed by quartile; thus, a model-based analysis was not conducted.

The rate of 28-day ACM was numerically higher in participants who did versus did not meet the ≥40% *ƒ*T > MIC target for imipenem (17% [*n* = 28/168] versus 11% [*n* = 4/37], respectively; Fig. 4). All participants with *K. pneumoniae* or *E. coli* met the target (Fig. S2). When considering participants with *P. aeruginosa* and *A. calcoaceticus–baumannii* complex, there was no substantial difference in 28-day ACM rates between the groups that did versus did not meet the imipenem exposure target (Fig. S2). A favorable response at EFU occurred in 61.9% (*n* = 104/168) of participants who achieved the imipenem exposure target, and 64.9% (*n* = 24/37) who did not achieve the target (Table 2).

## DISCUSSION

Potential relationships between imipenem and REL exposures measured via established PK/PD indices (21, 23) and select efficacy endpoints in the phase 3 RESTORE-IMI 2 study (11) were evaluated in this analysis. No meaningful trends between efficacy and imipenem %*ƒ*T > MIC and REL *f*AUC_0–24_/MIC were identified corresponding to exposures associated with the therapeutic dose(s) studied in this study. RESTORE-IMI 2 was an active controlled study in which only the therapeutic dose(s) for IMI/REL were studied. Therefore, the exposure–response relationship over a wide range of exposure (e.g., lower exposure associated with sub-therapeutic doses) could not be evaluated. Based on the totality of *in vitro* and *in vivo* data collected throughout the development of IMI/REL, it can be inferred that patients treated with IMI/REL show meaningfully better efficacy compared to patients not treated (PK concentration = 0 following administration of no intervention/placebo) or treated with sub-therapeutic doses of IMI/REL. Therefore, an overall exposure–efficacy relationship can be inferred over a wide range of exposure. However, since participants in the RESTORE-IMI-2 study were treated with only the therapeutic dose of IMI/REL, the majority of the study participants achieved the pre-specified PK/PD exposure target for both imipenem (~82%) and REL (~84%) (12, 13). Essentially, imipenem and REL exposures in this study are at the plateau of the exposure–efficacy relationship, confirming that the IMI/REL dose(s) studied in RESTORE-IMI 2 provide maximal target attainment and therapeutic antibacterial efficacy.

The pre-specified exposure indices for IMI/REL are a function of PK exposure and MIC values corresponding to pathogens. When the ACM rates and clinical response at EFU were stratified by baseline IMI/REL MIC values, a consistent and comparable trend of efficacy was observed across the range of MICs. This suggests that the IMI/REL dosing regimen studied in this study was sufficiently high to provide PK/PD target attainment across a wide range of MICs and/or pathogens.

In addition to assessing exposure–efficacy trend overall, evaluations were made by pathogen for four key pathogens. Most of the study participants achieved the pre-specified REL PK/PD target of *f*AUC_0–24_/MIC ≥ 8 when analyzed by *E. coli*, *K. pneumoniae*, and *P. aeruginosa*, while the majority of the participants did not achieve the target exposure for *A. calcoaceticus–baumannii* complex. However, for participants infected with *Acinetobacter*, ACM rates and clinical response rates were generally comparable between those who achieved the target and those who did not. Similar trends were observed in the case of imipenem. Since the goal of IMI/REL treatment is to achieve antibacterial activity for a wide range of target pathogens, conservative imipenem and REL PK/PD exposure targets were chosen. For difficult-to-treat pathogens, such as *Acinetobacter*, additional factors beyond exposure alone may influence antibacterial activity. It should also be noted that REL does not appear to enhance the antibacterial activity of imipenem for imipenem-non-susceptible *A. baumannii* isolates (7).

PK of both imipenem and REL are not affected by the intrinsic factors, such as age, sex, and race/ethnicity (21). ACM and clinical response at EFU were also similar across demographic subgroups (11). Therefore, no exposure–efficacy relationship is expected across any of these intrinsic factor subgroups.

The initial approval for the IMI/REL 500/500/250 mg dosing regimen in patients with cIAI and cUTI was based on the RESTORE-IMI 1 study (14); this dose level is also supported by a popPK model (21, 23). Dose adjustments to IMI/REL are recommended for patients with renal impairment, as the rate of creatinine clearance (CrCl) is the most influential covariate on the exposure of imipenem and REL (13, 23). Approved dosage adjustments in patients with renal impairment (12, 13) resulted in exposures that are expected to be similar to exposures for participants with normal renal function and suggest that no exposure–efficacy trend is expected in renally impaired patients (11, 21). Although patients with HABP/VABP may experience disease-associated changes in fluid distribution and renal function that could complicate IMI/REL dosing, an updated popPK model that included participants with HABP/VABP from the RESTORE-IMI 2 study did not change the key clinical pharmacology conclusions and supported use of the approved IMI/REL dosing regimen in patients with HABP/VABP (21). In addition, a recent sub-analysis from RESTORE-IMI 2 explored the efficacy, safety, and probability of target attainment of IMI/REL in adults with HABP/VABP and confirmed that participants with augmented renal clearance achieved sufficiently high drug exposures and favorable safety and efficacy profiles at approved dosing (24). Therefore, resulting PK exposures following the recommended and approved IMI/REL dosing regimen are expected to demonstrate equal achievement of the pre-specified PK targets for which there are close associations with antimicrobial activity overall and across intrinsic factor subgroups.

Similar findings regarding a lack of correlation between exposure and clinical efficacy in patients with HABP/VABP have been observed with other β-lactam/β-lactamase inhibitor combination agents, such as ceftolozane/tazobactam and ceftazidime/avibactam (25, 26). An exposure–efficacy analysis of the phase 3 ASPECT-NP study did not identify any consistent exposure–efficacy trends for ceftolozane or tazobactam, except at the highest MIC values with ceftolozane, for which an increase in 28-day ACM and a decrease in clinical cure at test of cure were detected. These results further supported the recommended ceftolozane/tazobactam dosing regimens for patients with HABP/VABP (25). PopPK modeling of a pooled analysis of phase 3 trials assessing patients with cIAI, cUTI, and HABP/VABP confirmed the final dosing recommendations of ceftazidime and avibactam, with CrCl being the only covariate to warrant dose adjustments (26).

The results presented here should not be generalized to other infection types or medical scenarios. One limitation of this analysis was the small size of the study population, which hindered the evaluation of the impact of covariates on the exposure parameters. With 211 participants providing PK data for REL and 205 for imipenem, the quartiles used to assess imipenem and REL exposure–efficacy trends contained data from small participant cohorts. Nevertheless, the fact that this analysis was conducted in the context of a phase 3 study, in participants with microbiologically confirmed HABP/VABP and utilizing stringent inclusion and exclusion criteria, strengthens the reliability of the findings while limiting the impact of potential confounding factors.

In conclusion, no trends were observed for 28-day ACM and clinical response at EFU with imipenem or REL exposure, which suggests an exposure–efficacy plateau in adult participants with HABP/VABP caused by gram-negative pathogens. These findings, based on phase 3 data from RESTORE-IMI 2, were consistent across the four key pathogens investigated and support the currently indicated IMI/REL 500/500/250 mg dosing regimen for patients with HABP/VABP.

## Data Availability

Merck Sharp & Dohme LLC, a subsidiary of Merck & Co., Inc., Rahway, New Jersey, USA (MSD) is committed to providing qualified scientific researchers access to anonymized data and clinical study reports from the company’s clinical trials for the purpose of conducting legitimate scientific research. MSD is also obligated to protect the rights and privacy of trial participants and, as such, has a procedure in place for evaluating and fulfilling requests for sharing company clinical trial data with qualified external scientific researchers. The MSD data sharing website (available at: https://externaldatasharing-msd.com/) outlines the process and requirements for submitting a data request. Applications will be promptly assessed for completeness and policy compliance. Feasible requests will be reviewed by a committee of MSD subject matter experts to assess the scientific validity of the request and the qualifications of the requestors. In line with data privacy legislation, submitters of approved requests must enter into a standard data-sharing agreement with MSD before data access is granted. Data will be made available for request after product approval in the US and EU or after product development is discontinued. There are circumstances that may prevent MSD from sharing requested data, including country or region-specific regulations. If the request is declined, it will be communicated to the investigator. Access to genetic or exploratory biomarker data requires a detailed, hypothesis-driven statistical analysis plan that is collaboratively developed by the requestor and MSD subject matter experts; after approval of the statistical analysis plan and execution of a data-sharing agreement, MSD will either perform the proposed analyses and share the results with the requestor or will construct biomarker covariates and add them to a file with clinical data that is uploaded to an analysis portal so that the requestor can perform the proposed analyses.

## SUPPLEMENTAL MATERIAL

Supplemental material aac.01313-25-s0001.docx Table S1; Fig. S1 and S2.

ASM does not own the copyrights to Supplemental Material that may be linked to, or accessed through, an article. The authors have granted ASM a non-exclusive, world-wide license to publish the Supplemental Material files. Please contact the corresponding author directly for reuse.

## References

[1] Candel FJ, Salavert M, Estella A, Ferrer M, Ferrer R, Gamazo JJ, García-Vidal C, Del Castillo JG, González-Ramallo VJ, Gordo F, Mirón-Rubio M, Pérez-Pallarés J, Pitart C, Del Pozo JL, Ramírez P, Rascado P, Reyes S, Ruiz-Garbajosa P, Suberviola B, Vidal P, Zaragoza R. 2023. Ten issues to update in nosocomial or hospital-acquired pneumonia: an expert review. J Clin Med 12:6526. doi:10.3390/jcm12206526 37892664 PMC10607368

[2] Karlowsky JA, Lob SH, Young K, Motyl MR, Sahm DF. 2021. Activity of ceftolozane/tazobactam against Gram-negative isolates from patients with lower respiratory tract infections – SMART United States 2018–2019. BMC Microbiol 21:74. doi:10.1186/s12866-021-02135-z 33676406 PMC7936229

[3] Micek ST, Wunderink RG, Kollef MH, Chen C, Rello J, Chastre J, Antonelli M, Welte T, Clair B, Ostermann H, Calbo E, Torres A, Menichetti F, Schramm GE, Menon V. 2015. An international multicenter retrospective study of Pseudomonas aeruginosa nosocomial pneumonia: impact of multidrug resistance. Crit Care 19:219. doi:10.1186/s13054-015-0926-5 25944081 PMC4446947

[4] Kollef MH, Nováček M, Kivistik Ü, Réa-Neto Á, Shime N, Martin-Loeches I, Timsit J-F, Wunderink RG, Bruno CJ, Huntington JA, Lin G, Yu B, Butterton JR, Rhee EG. 2019. Ceftolozane–tazobactam versus meropenem for treatment of nosocomial pneumonia (ASPECT-NP): a randomised, controlled, double-blind, phase 3, non-inferiority trial. Lancet Infect Dis 19:1299–1311. doi:10.1016/S1473-3099(19)30403-7 31563344

[5] Zilberberg MD, Shorr AF, Micek ST, Vazquez-Guillamet C, Kollef MH. 2014. Multi-drug resistance, inappropriate initial antibiotic therapy and mortality in Gram-negative severe sepsis and septic shock: a retrospective cohort study. Crit Care 18:596. doi:10.1186/s13054-014-0596-8 25412897 PMC4264255

[6] Zaragoza R, Vidal-Cortés P, Aguilar G, Borges M, Diaz E, Ferrer R, Maseda E, Nieto M, Nuvials FX, Ramirez P, Rodriguez A, Soriano C, Veganzones J, Martín-Loeches I. 2020. Update of the treatment of nosocomial pneumonia in the ICU. Crit Care 24:383. doi:10.1186/s13054-020-03091-2 32600375 PMC7322703

[7] Karlowsky JA, Lob SH, Kazmierczak KM, Hawser SP, Magnet S, Young K, Motyl MR, Sahm DF. 2018. In vitro activity of imipenem/relebactam against Gram-negative ESKAPE pathogens isolated in 17 European countries: 2015 SMART surveillance programme. J Antimicrob Chemother 73:1872–1879. doi:10.1093/jac/dky107 29659861

[8] Karlowsky JA, Lob SH, Kazmierczak KM, Young K, Motyl MR, Sahm DF. 2020. In vitro activity of imipenem/relebactam against Enterobacteriaceae and Pseudomonas aeruginosa isolated from intraabdominal and urinary tract infection samples: SMART surveillance United States 2015–2017. J Glob Antimicrob Resist 21:223–228. doi:10.1016/j.jgar.2019.10.028 31698105

[9] Karlowsky JA, Lob SH, Young K, Motyl MR, Sahm DF. 2021. In vitro activity of imipenem/relebactam against gram-negative bacilli from pediatric patients—Study for Monitoring Antimicrobial Resistance Trends (SMART) global surveillance program 2015–2017. J Pediatric Infect Dis Soc 10:274–281. doi:10.1093/jpids/piaa056 32535630

[10] Heo YA. 2021. Imipenem/cilastatin/relebactam: a review in gram-negative bacterial infections. Drugs 81:377–388. doi:10.1007/s40265-021-01471-8 33630278 PMC7905759

[11] Titov I, Wunderink RG, Roquilly A, Rodríguez Gonzalez D, David-Wang A, Boucher HW, Kaye KS, Losada MC, Du J, Tipping R, Rizk ML, Patel M, Brown ML, Young K, Kartsonis NA, Butterton JR, Paschke A, Chen LF. 2021. A randomized, double-blind, multicenter trial comparing efficacy and safety of imipenem/cilastatin/relebactam versus piperacillin/tazobactam in adults with hospital-acquired or ventilator-associated bacterial pneumonia (RESTORE-IMI 2 study). Clin Infect Dis 73:e4539–e4548. doi:10.1093/cid/ciaa803 32785589 PMC8662781

[12] European Medicines Agency. 2020. Recarbrio 500 mg/500 mg/250 mg powder for solution for infusion: summary of product characteristics. Available from: https://www.ema.europa.eu/en/documents/product-information/recarbrio-epar-product-information_en.pdf

[13] RECARBRIO (imipenem/cilastatin/relebactam). Prescribing Information. 2022. Rahway, NJ, USA Merck Sharp & Dohme LLC. https://www.accessdata.fda.gov/drugsatfda_docs/label/2020/212819s002lbl.pdf.

[14] Motsch J, Murta de Oliveira C, Stus V, Köksal I, Lyulko O, Boucher HW, Kaye KS, File TM Jr, Brown ML, Khan I, Du J, Joeng H-K, Tipping RW, Aggrey A, Young K, Kartsonis NA, Butterton JR, Paschke A. 2020. RESTORE-IMI 1: a multicenter, randomized, double-blind trial comparing efficacy and safety of imipenem/relebactam vs colistin plus imipenem in patients with imipenem-nonsusceptible bacterial infections. Clin Infect Dis 70:1799–1808. doi:10.1093/cid/ciz530 31400759 PMC7156774

[15] Leekha S, Terrell CL, Edson RS. 2011. General principles of antimicrobial therapy. Mayo Clin Proc 86:156–167. doi:10.4065/mcp.2010.0639 21282489 PMC3031442

[16] Ruiz-Ramos J, Gras-Martín L, Ramírez P. 2023. Antimicrobial pharmacokinetics and pharmacodynamics in critical care: adjusting the dose in extracorporeal circulation and to prevent the genesis of multiresistant bacteria. Antibiotics (Basel) 12:475. doi:10.3390/antibiotics12030475 36978342 PMC10044431

[17] Roberts JA, Abdul-Aziz MH, Lipman J, Mouton JW, Vinks AA, Felton TW, Hope WW, Farkas A, Neely MN, Schentag JJ, Drusano G, Frey OR, Theuretzbacher U, Kuti JL, International Society of Anti-Infective Pharmacology and the Pharmacokinetics and Pharmacodynamics Study Group of the European Society of Clinical Microbiology and Infectious Diseases. 2014. Individualised antibiotic dosing for patients who are critically ill: challenges and potential solutions. Lancet Infect Dis 14:498–509. doi:10.1016/S1473-3099(14)70036-2 24768475 PMC4181663

[18] Koyner JL, Murray PT. 2010. Mechanical ventilation and the kidney. Blood Purif 29:52–68. doi:10.1159/000259585 19923815 PMC2914396

[19] Roberts JA, Lipman J. 2009. Pharmacokinetic issues for antibiotics in the critically ill patient. Crit Care Med 37:840–851. doi:10.1097/CCM.0b013e3181961bff 19237886

[20] Levison ME, Levison JH. 2009. Pharmacokinetics and pharmacodynamics of antibacterial agents. Infect Dis Clin North Am 23:791–815. doi:10.1016/j.idc.2009.06.008 19909885 PMC3675903

[21] Patel M, Bellanti F, Daryani NM, Noormohamed N, Hilbert DW, Young K, Kulkarni P, Copalu W, Gheyas F, Rizk ML. 2022. Population pharmacokinetic/pharmacodynamic assessment of imipenem/cilastatin/relebactam in patients with hospital-acquired/ventilator-associated bacterial pneumonia. Clin Transl Sci 15:396–408. doi:10.1111/cts.13158 34704389 PMC8841461

[22] Rhee EG, Rizk ML, Calder N, Nefliu M, Warrington SJ, Schwartz MS, Mangin E, Boundy K, Bhagunde P, Colon-Gonzalez F, Jumes P, Liu Y, Butterton JR. 2018. Pharmacokinetics, safety, and tolerability of single and multiple doses of relebactam, a β-lactamase inhibitor, in combination with imipenem and cilastatin in healthy participants. Antimicrob Agents Chemother 62:e00280-18. doi:10.1128/AAC.00280-18 29914955 PMC6125551

[23] Bhagunde P, Patel P, Lala M, Watson K, Copalu W, Xu M, Kulkarni P, Young K, Rizk ML. 2019. Population pharmacokinetic analysis for imipenem-relebactam in healthy volunteers and patients with bacterial infections. CPT Pharmacometrics Syst Pharmacol 8:748–758. doi:10.1002/psp4.12462 31508899 PMC6813166

[24] Roberts JA, Nicolau DP, Martin-Loeches I, Deryke CA, Losada MC, Du J, Patel M, Rizk ML, Paschke A, Chen LF. 2023. Imipenem/cilastatin/relebactam efficacy, safety and probability of target attainment in adults with hospital-acquired or ventilator-associated bacterial pneumonia among patients with baseline renal impairment, normal renal function, and augmented renal clearance. JAC Antimicrob Resist 5:dlad011. doi:10.1093/jacamr/dlad011 36880088 PMC9985325

[25] Gao W, Passarell J, Patel YT, Zhang Z, Lin G, Fiedler-Kelly J, Bruno CJ, Rhee EG, De Anda CS, Feng H-P. 2022. Exposure-efficacy analyses support optimal dosing regimens of ceftolozane/tazobactam in participants with hospital-acquired/ventilator-associated bacterial pneumonia in ASPECT-NP. Antimicrob Agents Chemother 66:e0139921. doi:10.1128/aac.01399-21 35471040 PMC9112930

[26] Li J, Lovern M, Green ML, Chiu J, Zhou D, Comisar C, Xiong Y, Hing J, MacPherson M, Wright JG, Riccobene T, Carrothers TJ, Das S. 2019. Ceftazidime-avibactam population pharmacokinetic modeling and pharmacodynamic target attainment across adult indications and patient subgroups. Clin Transl Sci 12:151–163. doi:10.1111/cts.12585 30221827 PMC6440567

