# Supplementary material for: Imipenem/cilastatin/relebactam dosing regimen justification using exposure–efficacy analyses in participants with hospital-acquired bacterial pneumonia or ventilator-associated bacterial pneumonia in the RESTORE-IMI 2 phase 3 study
Source: Antimicrob Agents Chemother. 2026 Jan 26;70(3):e01313-25. doi: 10.1128/aac.01313-25 (PMC12959156; doi:10.1128/aac.01313-25)
Supplement: Supplemental material — Table S1; Fig. S1 and S2. [file aac.01313-25-s0001.docx]

Table S1 Summary of efficacy endpoints by baseline imipenem/REL MIC in the exposure-efficacy population.

| **MIC, µg/mL** | **28-day ACM,  *n*/*N* (%)** | **Cure at EFU, *n*/*N* (%)** |
| --- | --- | --- |
| 0.03 | 1/11 (9.1) | 7/11 (63.6) |
| 0.06 | 2/15 (13.3) | 10/15 (66.7) |
| 0.12 | 7/45 (15.6) | 29/45 (64.4) |
| 0.25 | 6/46 (13.0) | 30/46 (65.2) |
| 0.5 | 8/23 (34.8) | 11/23 (47.8) |
| 1 | 2/13 (15.4) | 6/13 (46.2) |
| 2 | 3/10 (30.0) | 5/10 (50.0) |
| 4 | 0/5 (0) | 5/5 (100.0) |
| 8 | 0/1 (0) | 1/1 (100.0) |
| 16 | 0/4 (0) | 4/4 (100) |
| 32 | 5/38 (13.2) | 23/38 (60.5) |

ACM, all-cause mortality; EFU, early follow-up; MIC, minimum inhibitory concentration; REL, relebactam.

FIG S1 All-cause mortality by achievement of the relebactam exposure target
(*f*AUC_0–24_/MIC ≥ 8), and each key pathogen.


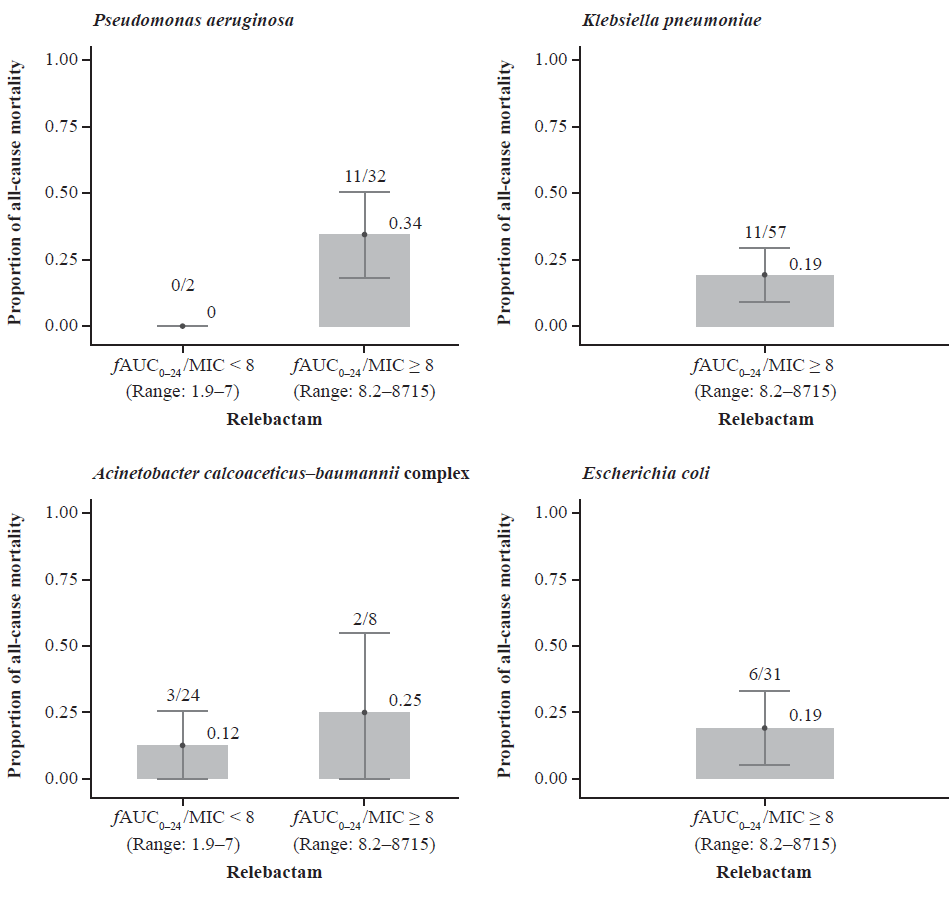


FIG S2 All-cause mortality by achievement of target imipenem exposure (*f*T > MIC for at least 40% of the dosing interval) and each key pathogen.


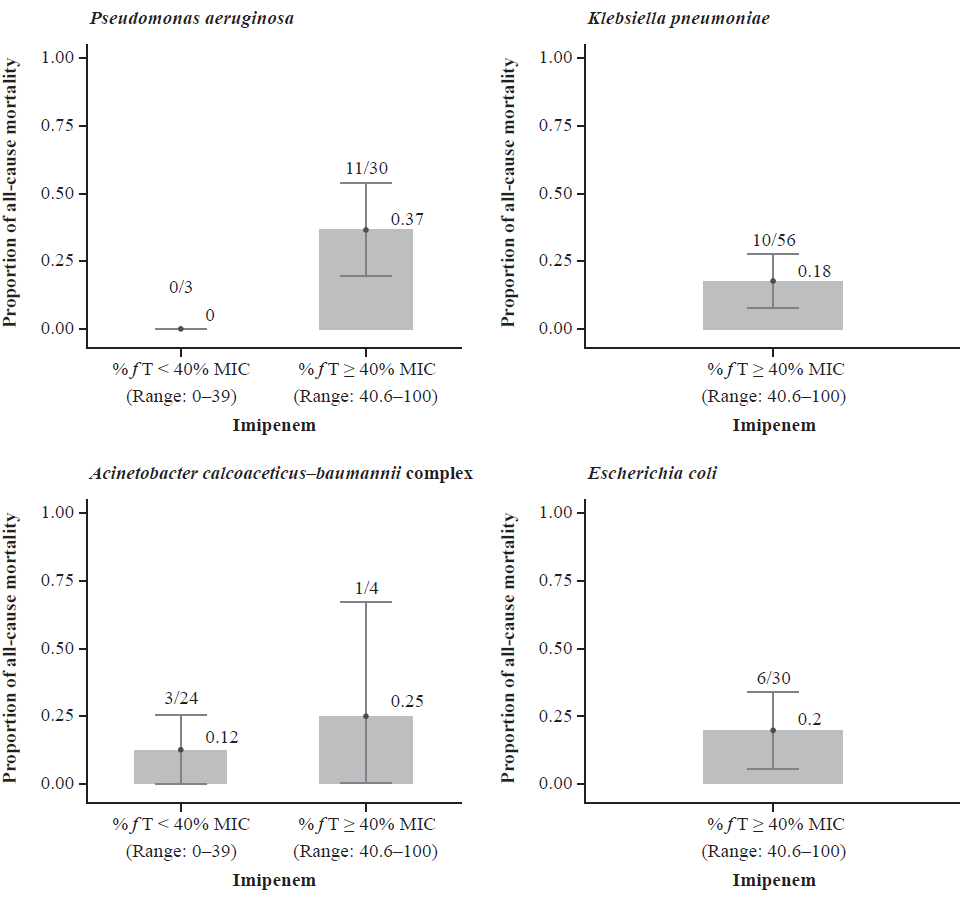


No participants with exposure below the target (%*f*T > MIC for at least 40% of the dosing interval) had *Klebsiella pneumoniae* or *Escherichia coli* infection.
%*f*T > MIC, percentage of time free drug concentration exceeded the minimum inhibitory concentration.
